# A Component-Level Network Analysis of Emotional Symptoms, Sleep Problems, and Physical Activity Identity at a Chinese University

**DOI:** 10.3390/bs16101751

**Published:** 2026-09-25

**Authors:** Yazhuo Zhang, Jieyu Chen, Weida Dong

**Affiliations:** 1 Department of Public Physical and Art Education, Zhejiang University, Hangzhou 310000, China; 2 Physical Education College, Jilin University, Changchun 130000, China; chenjy0522@mails.jlu.edu.cn

**Keywords:** component-level network analysis, university students, depression, anxiety, stress, sleep problems, physical activity identity, sex differences

## Abstract

This cross-sectional study estimated a component-level network of depression, anxiety, and stress dimensions, sleep components, and physical activity identity among students at one Chinese university. Using convenience and snowball sampling, 3762 students at Zhejiang University completed the DASS-21, PSQI, and MIPAI-25. Regularized partial-correlation networks were estimated with EBICglasso (gamma = 0.50) using automatic correlation selection. Strength, bridge strength, signed bridge expected influence, and predictability were calculated. Network accuracy and stability were examined with 1000 bootstrap resamples, and sex differences were assessed using a Network Comparison Test with 1000 permutations. Scores above the prespecified screening cutoffs were observed for depression (76.0%), anxiety (79.5%), stress (62.8%), and poor sleep quality (80.2%). In the DASS-PSQI network, hypnotic medication use had the largest overall strength point estimate (1.126), whereas depression and anxiety subscales had the largest bridge-strength point estimates (0.121 and 0.093). In the exploratory three-domain network, hypnotic medication use again had the largest strength estimate (1.131); MIPAI acknowledgment had the highest bridge-strength point estimate (0.134), closely followed by depression (0.130), and remained the highest-ranked MIPAI dimension in the mixed graphical model. Its individual cross-domain edges were small, and their specific pattern was model-dependent. Overall network structure did not differ significantly by sex (M = 0.087, *p* = 0.117), whereas global strength was higher in males than females (7.11 vs. 6.83; *p* = 0.012); no individual edge remained significant after Holm correction. These findings describe cross-sectional conditional associations and do not establish causal or protective effects.

## 1. Introduction

Mental health and sleep problems are common among university students and are associated with impaired academic functioning, social adaptation, and well-being. International surveys suggest that approximately one-third of first-year students meet criteria for at least one common mental disorder (Auerbach et al., 2018), while a meta-analysis reported a depression prevalence of 34.70% among Chinese college students (Li et al., 2022). Sleep problems are also common in this population (King et al., 2023). These findings support examining emotional and sleep-related dimensions together rather than as isolated outcomes.

Depression, anxiety, stress, and sleep disturbances show substantial comorbidity and prospective associations (Baldwin et al., 2002; Hertenstein et al., 2019; Madani et al., 2010). Sleep problems may precede or accompany emotional distress, while emotional distress may also be associated with subsequent sleep difficulties. Because the present study is cross-sectional, however, its network estimates represent conditional associations at one time point and cannot determine temporal direction or causal pathways.

The functional and health consequences associated with emotional and sleep problems make it important to identify which measured dimensions are most strongly connected within the observed system (Rice & Miller, 1998; Silva et al., 2015; Toro et al., 2025). Such information can generate hypotheses for longitudinal and intervention research, but it should not be interpreted as evidence that a central node is a causal driver or an established treatment target.

Traditional analyses based only on total scale scores can obscure differences among subscales or components. Network analysis represents measured variables as nodes and conditional associations as edges, allowing investigators to describe connectivity and cross-domain bridge patterns (Borsboom, 2017; Cai et al., 2024). In the present study, the nodes are aggregated DASS subscales, PSQI components, and MIPAI dimensions; therefore, the analysis is a component- or dimension-level network rather than an item- or symptom-level network. This level of analysis offers more resolution than total-score correlations but does not identify individual symptom relations.

Physical activity is associated with mental health and sleep outcomes, but behavior and identity are conceptually distinct (Carpenter et al., 2021; Tutakhail et al., 2026). Physical activity identity refers to the extent to which being physically active is incorporated into the self and, in the MIPAI-25, comprises acknowledgment, compatibility, centrality, commitment, and exploration (Brown et al., 2026). Acknowledgment reflects recognition by oneself and others as a physically active person. Although identity may be associated with sustained activity, a cross-sectional network cannot establish that any identity dimension is protective or that changing it would improve emotional or sleep outcomes.

Sex-related differences have been reported in emotional symptoms, sleep, and physical activity-related self-concept (Sanfilippo et al., 2024). The present dataset records participants as male or female; accordingly, the term sex is used consistently. Network comparisons can describe whether global strength or the pattern of estimated edges differs between these groups, but such differences may also reflect group differences in score distributions, variances, or measurement properties.

Previous network studies have commonly focused on emotional variables or sleep outcomes without jointly incorporating a multidimensional measure of physical activity identity (Luo et al., 2025). In addition, many analyses have been conducted at either the total-score or item level, leaving the component-level organization of these domains less clearly described. The present study addresses this descriptive gap while explicitly limiting inference to aggregated dimensions measured in a single-university sample.

This study therefore aimed to (1) estimate a DASS-PSQI component-level network, (2) examine how the network changed descriptively after adding five MIPAI dimensions, (3) identify nodes with the largest strength and bridge-strength point estimates, and (4) explore sex-based network differences. The analyses were primarily exploratory. Based on the prior literature, depression and anxiety were expected to be highly connected within the DASS-PSQI network, while no directional or causal hypothesis was made for the MIPAI dimensions.

## 2. Materials and Methods

### 2.1. Study Design and Participants

This cross-sectional survey was conducted at Zhejiang University, Zhejiang Province, China, between October and November 2025. Students were recruited through convenience and snowball sampling. Initial recruitment occurred in physical education classes taught by members of the research team, after which invitations were shared through WeChat and Tencent QQ. This open-link recruitment approach did not provide a defined sampling frame or a total count of invitation recipients. Participation was voluntary and anonymous; for students recruited from physical education classes, participation or refusal did not affect course grades, attendance records, or any other academic assessment. Data were collected using the web-based version of the Wenjuanxing platform(Changsha Ranxing Information Technology Co., Ltd., Changsha, China).

A total of 3984 students started the questionnaire. During quality control, 222 questionnaires were excluded: 169 because of missing items, 32 because of implausibly short completion times, and 21 because of logically inconsistent responses. These classifications were assigned during the original quality-control process; however, the exact elapsed-time cutoff and detailed inconsistency rules were not retained in the archived analytic files and could not be reconstructed retrospectively. The final analytic sample therefore comprised 3762 complete responses, corresponding to a valid-completion proportion of 94.43% among the started questionnaires. Before accessing the questionnaire, all participants reviewed an electronic informed consent statement and indicated consent to proceed.

### 2.2. Measurement Instruments

The following instruments assessed negative emotional symptoms, sleep problems, and physical activity identity. Item-to-component mappings are provided in Table S1. Internal consistency in the present analytic sample was evaluated using Cronbach’s alpha.

### 2.3. Depression, Anxiety, and Stress Symptoms

Depression, anxiety, and stress symptoms were assessed with a simplified-Chinese DASS-21 questionnaire. Psychometric support for a simplified-Chinese DASS-21 has been reported in mainland Chinese university students (K. Wang et al., 2016). The retained study materials did not document the translator or exact source edition of the questionnaire administered; consequently, we cite the validation evidence for the Chinese-language instrument but do not claim that the administered wording was taken from that specific published version. Each item is rated from 0 to 3. Seven items were summed for each raw subscale, producing observed and theoretical ranges of 0–21: DASS1 (depression; items 3, 5, 10, 13, 16, 17, and 21), DASS2 (anxiety; items 2, 4, 7, 9, 15, 19, and 20), and DASS3 (stress; items 1, 6, 8, 11, 12, 14, and 18). Scores were not multiplied by two for network estimation or descriptive statistics. For screening summaries, raw-score cutoffs equivalent to the conventional doubled-score thresholds were used: depression >= 5, anxiety >= 4, and stress >= 8 (Lovibond & Lovibond, 1995). These classifications indicate scores above screening cutoffs and are not clinical diagnoses. Cronbach’s alpha was 0.934, 0.931, and 0.929 for the depression, anxiety, and stress subscales, respectively (total DASS-21 alpha = 0.976).

### 2.4. Sleep Problems

Sleep problems were assessed with a Chinese-language Pittsburgh Sleep Quality Index (PSQI), covering the preceding month. Chinese PSQI versions have demonstrated reliability and validity in Chinese populations, including university medical students (Tsai et al., 2005; Zheng et al., 2016). The retained study materials did not document the translator or exact source edition used; therefore, these references are cited as psychometric support for Chinese-language PSQI administration rather than as confirmation of the exact wording administered. Nineteen self-rated items are combined into seven components scored from 0 to 3: subjective sleep quality (PSQI1), sleep latency (PSQI2), sleep duration (PSQI3), habitual sleep efficiency (PSQI4), sleep disturbances (PSQI5), hypnotic medication use (PSQI6), and daytime dysfunction (PSQI7). The seven components served as network nodes and summed to a global score ranging from 0 to 21. A global PSQI score > 7 was classified as poor sleep quality. Cronbach’s alpha across the seven component scores was 0.756. Because sleep latency, duration, and efficiency share information in their scoring, edges among PSQI2–PSQI4 were interpreted cautiously.

### 2.5. Physical Activity Identity

Physical activity identity was assessed with the 25-item Multidimensional Inventory of Physical Activity Identity (MIPAI-25), originally developed and validated by Brown et al. (2026). No independently validated Chinese version was available to our knowledge. A study-specific Chinese wording was prepared internally by the research team. No formal back-translation, independent expert review, cognitive interviewing, or pilot testing was conducted. A separately documented written permission to translate and use the instrument could not be confirmed from the retained study records. The Chinese wording should therefore be regarded as an unvalidated study-specific translation, and its measurement properties beyond internal consistency remain uncertain. Items are rated on a 7-point scale and summed into five 5-item dimensions: acknowledgment (MIPAI1), compatibility (MIPAI2), centrality (MIPAI3), commitment (MIPAI4), and exploration (MIPAI5), each ranging from 5 to 35. Cronbach’s alpha was 0.937, 0.930, 0.927, 0.930, and 0.931 for MIPAI1–MIPAI5, respectively (total MIPAI-25 alpha = 0.985).

### 2.6. Statistical Analysis

Analyses were conducted in R 4.3.2(R Core Team, R Foundation for Statistical Computing, Vienna, Austria). The principal packages were bootnet 1.8, qgraph 1.9.8, networktools 1.6.0, mgm 1.2-15, and NetworkComparisonTest 2.2.2. All 3762 analytic records were complete for the network variables; no imputation was performed. Means, standard deviations, skewness, and kurtosis were calculated for the three DASS subscales, seven PSQI components, and five MIPAI dimensions. Conventional *p* values were used for permutation tests and bootstrap difference tests where applicable; an EBICglasso non-zero edge was not interpreted as the result of a conventional null-hypothesis significance test.

### 2.7. Network Construction

Two regularized partial-correlation networks were estimated with bootnet::estimateNetwork using EBICglasso. The first included the three DASS subscales and seven PSQI components; the second added the five MIPAI dimensions. The input association matrix was selected with qgraph::cor_auto: the seven four-level PSQI components were detected as ordinal, while DASS and MIPAI summed scores were treated as continuous, yielding polychoric, polyserial, or Pearson associations as appropriate. The EBIC tuning parameter was gamma = 0.50. Edges represent conditional associations after controlling for all remaining nodes. Blue solid edges indicate positive associations, red dashed edges indicate negative associations, and edge width represents absolute magnitude. Because a dense regularized solution was selected, the smallest non-zero edges were interpreted cautiously.

As a sensitivity analysis, a mixed graphical model was estimated with mgm 1.2-15, treating DASS and MIPAI summed scores as Gaussian variables and the seven PSQI components as four-level categorical variables. Regularization was selected by EBIC (lambdaGam = 0.25) with the OR rule. Agreement with the primary network was summarized using Spearman correlations of absolute edge weights, strength ranks, and bridge-strength ranks, together with the number of non-zero edges. Absolute edge weights were used because signs are not consistently defined for all mixed-variable MGM interactions. Communities were prespecified by instrument/domain (DASS, PSQI, and MIPAI).

A post hoc simulation-based sensitivity analysis was conducted to describe expected network-estimation performance rather than to provide an a priori sample-size justification or claim a formal power threshold. The estimated 15-node network was used as the data-generating network; ordinal data with five levels were simulated for sample sizes of 800, 1200, 1600, 2000, and 2400, with 500 replications per condition. Because this simulation used a different tuning setting from the primary model and was conducted after data collection, it is presented only as a descriptive supplementary analysis (Figure S1) and is not used to establish sample adequacy. The post hoc simulation was conducted using the netSimulator function in the bootnet package, with EBICglasso and an EBIC tuning parameter of gamma = 0.50. Sensitivity represented the proportion of non-zero edges in the data-generating network that were recovered, whereas specificity represented the proportion of zero edges that remained absent.

### 2.8. Centrality and Predictability Analysis

Strength was calculated as the sum of the absolute weights of all edges incident on each node. Bridge strength and signed one-step and two-step bridge expected influence were calculated using the bridge function in the networktools package (Jones et al., 2021). Communities were prespecified by instrument domain, comprising DASS and PSQI in the two-domain network and DASS, PSQI, and MIPAI in the three-domain network. Complete one-step and two-step signed bridge expected influence values are reported in Table S2a,b. Because two-step values incorporate indirect paths, they were treated as supplementary descriptive metrics. Predictability was estimated using the predict method in the mgm package and expressed as R2, representing the proportion of variance explained by adjacent nodes (Haslbeck & Waldorp, 2018). Because this predictability model treated all aggregated/component scores as Gaussian, predictability values were interpreted descriptively. Centrality and bridge metrics quantify connectivity, not causal influence, clinical importance, or protective effects.

### 2.9. Network Stability and Accuracy Assessment

Network accuracy and stability were evaluated using the bootnet function in the bootnet package (Epskamp et al., 2018), with 1000 nonparametric bootstrap resamples for edge-weight confidence intervals and difference tests and 1000 case-dropping bootstrap resamples for centrality stability. Correlation-stability (CS) coefficients above 0.25 were considered acceptable and values above 0.50 preferable. For the edge-weight CS analysis, the vector of unique edge estimates from each case-dropping bootstrap network was correlated with the corresponding full-sample edge-weight vector. The CS coefficient represented the maximum proportion of cases that could be dropped while retaining a correlation of at least 0.70 in 95% of bootstrap samples. This edge-weight stability analysis was distinct from edge-weight accuracy, which was assessed using nonparametric bootstrap confidence intervals. Difference plots were used to assess whether the largest point estimates were statistically distinguishable from other estimates; numerical rank alone was not interpreted as evidence of a uniquely dominant node.

### 2.10. Sex-Stratified Network Comparison

Male and female networks were compared using the NCT function in the NetworkComparisonTest package (van Borkulo et al., 2023), with gamma = 0.50 and 1000 permutations. Tests examined global strength, maximum structural difference, individual edges, and strength centrality. Edgewise multiplicity was controlled using the Holm method. An exploratory residence-based comparison (rural vs. urban) used the same settings. Because formal measurement invariance across sex was not evaluated in the supplied output, the sex-based NCT was interpreted as exploratory.

## 3. Results

### 3.1. Participant Characteristics

The mean raw scores for DASS depression, anxiety, and stress were 10.19 (SD = 6.43), 10.29 (SD = 6.37), and 10.36 (SD = 6.30), respectively. Scores were above the screening cutoffs for depression in 2859 students (76.0%), anxiety in 2991 (79.5%), and stress in 2363 (62.8%). The mean DASS total was 30.84 (SD = 18.67). The mean PSQI global score was 11.62 (SD = 4.45), and 3019 students (80.2%) had PSQI scores > 7. The mean MIPAI total was 111.26 (SD = 36.79) (Table 1). Across the 15 network nodes, skewness ranged from −0.354 to 0.066 and kurtosis from −1.452 to −0.434 (Table S2).

### 3.2. DASS-PSQI Component-Level Network and Bridge Metrics

Figure 1 shows the DASS-PSQI regularized partial-correlation network. The largest within-domain edges were PSQI2–PSQI5 (0.54), PSQI3–PSQI4 (0.52), DASS1–DASS3 (0.49), DASS2–DASS3 (0.47), and DASS1–DASS2 (0.46). The largest overall strength point estimate was observed for PSQI6 (hypnotic medication use; 1.126), followed by DASS1 (depression; 1.070) and DASS2 (anxiety; 1.022). Predictability was highest for DASS1 (R2 = 0.902), DASS3 (0.900), and DASS2 (0.898). Among PSQI components, predictability ranged from 0.280 for PSQI4 to 0.525 for PSQI6 (Table S2a).

Figure 2 presents standardized bridge-strength estimates for the DASS-PSQI network. DASS1 had the largest bridge-strength point estimate (0.121), followed by DASS2 (0.093), PSQI5 (sleep disturbance; 0.083), and PSQI6 (0.075). DASS3 had a smaller bridge-strength estimate (0.018), and PSQI7 was close to zero (0.001). The CS coefficients for overall strength and bridge strength were both 0.750. The CS coefficient was also 0.750 for closeness but was lower for betweenness (0.439). Bootstrap difference plots should be consulted before interpreting numerical ranks as statistically distinct (Figure S3).

### 3.3. DASS-PSQI-MIPAI Component-Level Network and Bridge Metrics

Figure 3 shows the regularized partial-correlation network after the five MIPAI dimensions were added. The strongest edges remained within domains: PSQI2–PSQI5 (0.54), PSQI3–PSQI4 (0.52), DASS1–DASS3 (0.49), DASS2–DASS3 (0.47), and DASS1-DASS2 (0.46). Within the MIPAI domain, the largest edges were MIPAI2–MIPAI4 (0.36), MIPAI1–MIPAI3 (0.35), and MIPAI4–MIPAI5 (0.26). Direct MIPAI-DASS and MIPAI-PSQI edges were small in absolute magnitude.

In the three-domain network, PSQI6 had the largest strength point estimate (1.131), followed by DASS1 (1.078), MIPAI1 (1.057), MIPAI2 (1.056), MIPAI4 (1.030), and MIPAI3 (1.024). These estimates were close in magnitude and were not treated as evidence of a uniquely central node. Predictability was highest for MIPAI3 (R^2^ = 0.913), MIPAI2 (0.912), and MIPAI4 (0.910), and lowest for PSQI4 (0.283) and PSQI3 (0.294) (Table S2b).

Figure 4 presents standardized bridge-strength estimates for the three-domain network. MIPAI1 (acknowledgment) had the largest bridge-strength point estimate (0.134), closely followed by DASS1 (0.130), PSQI5 (0.116), DASS2 (0.093), and PSQI6 (0.090). MIPAI1 also had a positive one-step bridge expected influence of 0.134. In contrast, MIPAI2 and MIPAI4 had negative signed one-step bridge expected influence (−0.048 and −0.028), despite positive bridge-strength values based on absolute edges. The largest cross-community edge was DASS1–PSQI6 (0.038). Among the cross-community edges involving MIPAI, the largest was PSQI4–MIPAI1 (0.031 after rounding; Table S5). Because bridge strength is based on absolute edge weights, it does not retain the direction of individual cross-domain associations.

For the three-domain network, the case-dropping CS coefficients for strength, bridge strength, closeness, and the vector of edge estimates were each 0.750; the coefficient for betweenness was 0.205. The edge-weight CS coefficient summarizes the stability of the complete edge-weight vector under case dropping and is distinct from edge accuracy, which was assessed using nonparametric bootstrap confidence intervals. The bootstrap confidence intervals and high CS coefficients for the primary metrics supported estimation stability, while the lower betweenness stability supported excluding strong interpretations of that index (Figure S7). Bootstrap difference tests indicated that several centrality estimates were not clearly distinguishable; therefore, results are described as point-estimate rankings.

The mixed graphical model sensitivity analysis retained 86 non-zero edges compared with 60 in the primary EBICglasso network. Agreement with the primary network was higher for absolute edge weights (Spearman rho = 0.727) and moderate for bridge-strength ranks (rho = 0.489), whereas strength-rank agreement was weak and negative (rho = −0.293; Table S6). Among the MIPAI dimensions, MIPAI1 retained the largest MGM bridge-strength point estimate (bridge strength = 0.390). The non-zero MIPAI1 cross-domain edges retained in the MGM were MIPAI1–PSQI2 (0.082), MIPAI1–PSQI1 (0.062), MIPAI1–PSQI7 (0.057), MIPAI1–PSQI3 (0.052), MIPAI1–PSQI6 (0.050), MIPAI1–PSQI4 (0.040), MIPAI1–PSQI5 (0.038), and MIPAI1–DASS1 (0.008). These results indicate that MIPAI1 remained the most bridge-connected MIPAI dimension in the MGM sensitivity model, although the number and distribution of retained edges differed from the primary EBICglasso network.

### 3.4. Sex-Based Comparison of the Three-Domain Network

The male (n = 1752) and female (n = 2010) groups had mean total scores of 32.02 versus 29.81 for DASS, 11.77 versus 11.49 for PSQI, and 113.48 versus 109.32 for MIPAI, respectively. The Network Comparison Test showed no statistically significant difference in overall network structure (M = 0.087, *p* = 0.117). Global strength was higher in the male network than in the female network (7.111 vs. 6.832; S = 0.279, *p* = 0.012) (Figure 5). No individual edge or node-strength difference remained significant after Holm correction. An exploratory rural-versus-urban comparison also found no difference in overall structure (M = 0.056, *p* = 0.853) or global strength (6.868 vs. 7.025; *p* = 0.162; Figure S8). Sex-specific means and standard deviations for all 15 network variables are reported in Table S7; these descriptive differences may contribute to the observed global-strength difference.

## 4. Discussion

This component-level network analysis identified several highly connected DASS and PSQI dimensions and described how five physical activity identity dimensions were positioned in an expanded network. In the two-domain network, PSQI6 had the largest strength point estimate, and DASS1 and DASS2 had the largest bridge-strength estimates. In the three-domain network, MIPAI1 had the largest bridge-strength point estimate in the primary model, although most MIPAI cross-domain edges were small. The sex-stratified networks did not differ significantly in overall structure, but global strength was higher in males. These findings describe connectivity among aggregated dimensions at one assessment and do not establish temporal, causal, protective, or intervention effects.

In the DASS-PSQI network, PSQI6 had the largest overall strength point estimate, followed by DASS1 and DASS2. Overall strength reflects a node’s total absolute connectivity within the network. By contrast, DASS1 and DASS2 had the largest bridge-strength point estimates, indicating comparatively greater absolute connectivity with PSQI components. Among the PSQI components, PSQI5 and PSQI6 had the largest bridge-strength point estimates, while PSQI7 had a bridge-strength estimate close to zero. These rankings are point estimates and should not be interpreted as evidence that any dimension was uniquely dominant without support from bootstrap difference tests. The relatively large bridge-strength estimates for DASS1 and DASS2 indicate that the depression and anxiety subscales had greater cross-domain connectivity with PSQI components than the stress subscale. This pattern is consistent with previous research showing close associations among depression, anxiety, and sleep problems (Kaiser et al., 2021; Medina et al., 2014; T. Wang et al., 2023; Zou et al., 2024). Because these data were collected at a single time point, the direction of these associations remains unknown.

PSQI6 was highly connected in the observed networks, but this component measures only the reported frequency of hypnotic medication use during the preceding month. It does not identify medication type, dose, indication, treatment duration, dependence, adverse effects, or the temporal relationship between medication use and other dimensions. The present results therefore cannot support conclusions about pharmacological dependence, medication-related deterioration, or the relative priority of pharmacological and non-pharmacological interventions. Although previous studies have examined hypnotic medication use and daytime functioning in relation to mental health (Fava, 2004; M. Wang et al., 2023; Zhang et al., 2026), PSQI7 showed little cross-domain connectivity in this sample. This contrast further illustrates why interpretations must follow the present component-level estimates rather than broader symptom-level assumptions.

Associations involving sleep latency, sleep duration, and habitual sleep efficiency should be interpreted in light of the PSQI scoring structure. The scoring algorithms for these components use related sleep-timing information, and their associations may therefore partly reflect measurement overlap. Because we did not conduct a sensitivity analysis excluding the overlapping components, the extent to which this overlap influenced the estimated edges remains unknown. Accordingly, associations involving these components, particularly the PSQI3–PSQI4 edge, should be interpreted cautiously.

The MIPAI findings should be regarded as exploratory because they were obtained using a study-specific Chinese translation without formal back-translation, independent expert review, cognitive interviewing, pilot testing, or independent Chinese psychometric validation. In the primary three-domain network, MIPAI1 had the largest bridge-strength point estimate, closely followed by DASS1. However, all direct MIPAI-DASS and MIPAI-PSQI edges had absolute weights of 0.031 or less, and stronger MIPAI associations occurred within the identity domain. Bridge strength sums absolute cross-domain edges and does not retain their direction. Although MIPAI1 had a positive signed one-step bridge expected influence, this statistic does not establish a psychological mechanism or protective role. Previous studies have reported associations between physical activity identity, physical activity, and affective outcomes (Forshaw et al., 2023; Shi et al., 2024), but actual physical activity behavior was not measured here. The current study therefore cannot compare the relative importance of identity and behavior or identify MIPAI1 as an intervention target.

The mixed graphical model sensitivity analysis indicated that MIPAI1 retained the largest bridge-strength estimate among the MIPAI dimensions, with eight non-zero cross-domain edges, primarily involving PSQI components. However, the sensitivity model differed from the primary EBICglasso network in the number and distribution of retained edges, and strength-rank agreement was weak. Therefore, the MIPAI1 finding should be interpreted as a descriptive robustness check rather than evidence of a stable intervention target or causal mechanism.

The sex-based comparison was also exploratory. Overall network structure did not differ significantly between males and females, and global strength was higher in males than females. No individual edge or node-strength difference remained significant after Holm correction. The global-strength result indicates a difference in the sum of absolute edge weights, but its practical meaning is uncertain. Differences in variances, marginal score distributions, or measurement properties could contribute to the result. Previous network studies have reported sex-related differences in affective and sleep variables (Liu et al., 2025; Su et al., 2024), but the present study did not assess emotional suppression, help-seeking, social support, or gender-role socialization. These unmeasured factors cannot explain the observed result. Furthermore, the non-significant structure test does not demonstrate network equivalence or universal applicability of a common intervention framework. Formal measurement invariance should be established before stronger sex-based interpretations are considered.

Strengths of this study include its large analytic sample; the clear and transparent separation of the two-domain and three-domain network analyses; the reporting of strength, bridge strength, signed bridge expected influence, and predictability; and the bootstrap-based assessment of network accuracy and stability.Exact CS coefficients and corrected network-specific tables improve reproducibility. The interpretation distinguishes point-estimate rankings from statistically established differences and avoids equating connectivity with causality or clinical importance.

Several limitations require emphasis. First, the cross-sectional design and reliance on self-reported measures preclude temporal or causal inference, and physical activity behavior was not directly assessed. Second, recruitment through convenience and snowball sampling at one university may have introduced selection bias and limits generalizability. Complete-case exclusions may have further affected the analytic sample. Although reason-specific counts were recovered (169 missing-item, 32 short-completion, and 21 logically inconsistent questionnaires), the exact completion-time cutoff and detailed inconsistency rules were not retained, limiting reproducibility of the original quality-control process. The reported 94.43% represents a valid-completion proportion among the started questionnaires rather than a population response rate. Third, the study-specific Chinese MIPAI-25 translation lacked formal translation and independent validation, and separately documented written permission to translate and use the instrument could not be confirmed from the retained study records; the MIPAI findings therefore remain exploratory. Fourth, the aggregation of DASS and MIPAI items reduced item-level resolution. In addition, the potential influence of overlapping PSQI component scoring was not examined through a component-exclusion sensitivity analysis. Finally, sex-based comparisons should be interpreted cautiously because measurement invariance was not established.

Future studies should replicate these component-level networks in multi-university samples and use a formally translated and validated Chinese MIPAI-25. Longitudinal research is needed to examine temporal ordering, and experimental studies are required before intervention effects can be evaluated. Future analyses should also assess measurement invariance, report node-level mixed graphical model results, and test the influence of overlapping PSQI components. Item-level analyses could additionally evaluate whether the aggregated DASS and MIPAI patterns conceal heterogeneous relations among individual items.

## 5. Conclusions

In this single-university, cross-sectional sample, component-level networks described conditional associations among DASS subscales, PSQI components, and MIPAI dimensions. Hypnotic medication use had the largest strength point estimate, depression and anxiety had the largest DASS-PSQI bridge-strength estimates, and MIPAI acknowledgment had the largest bridge-strength point estimate after the MIPAI dimensions were added. Most direct MIPAI cross-domain edges were small, and the sensitivity results indicated that the smallest edges depended on how mixed measurement scales were modeled. MIPAI acknowledgment remained the highest-ranked MIPAI dimension across models, although its specific cross-domain edge pattern varied by model specification. The cross-sectional design precludes causal or protective interpretations. Male and female networks showed no statistically detectable difference in overall structure, although global strength was higher in males. Generalization is limited to student populations with institutional and sampling characteristics similar to this single-university sample. These findings generate hypotheses requiring replication, measurement-invariance testing, and longitudinal or experimental evaluation.

## Figures and Tables

**Figure 1:**
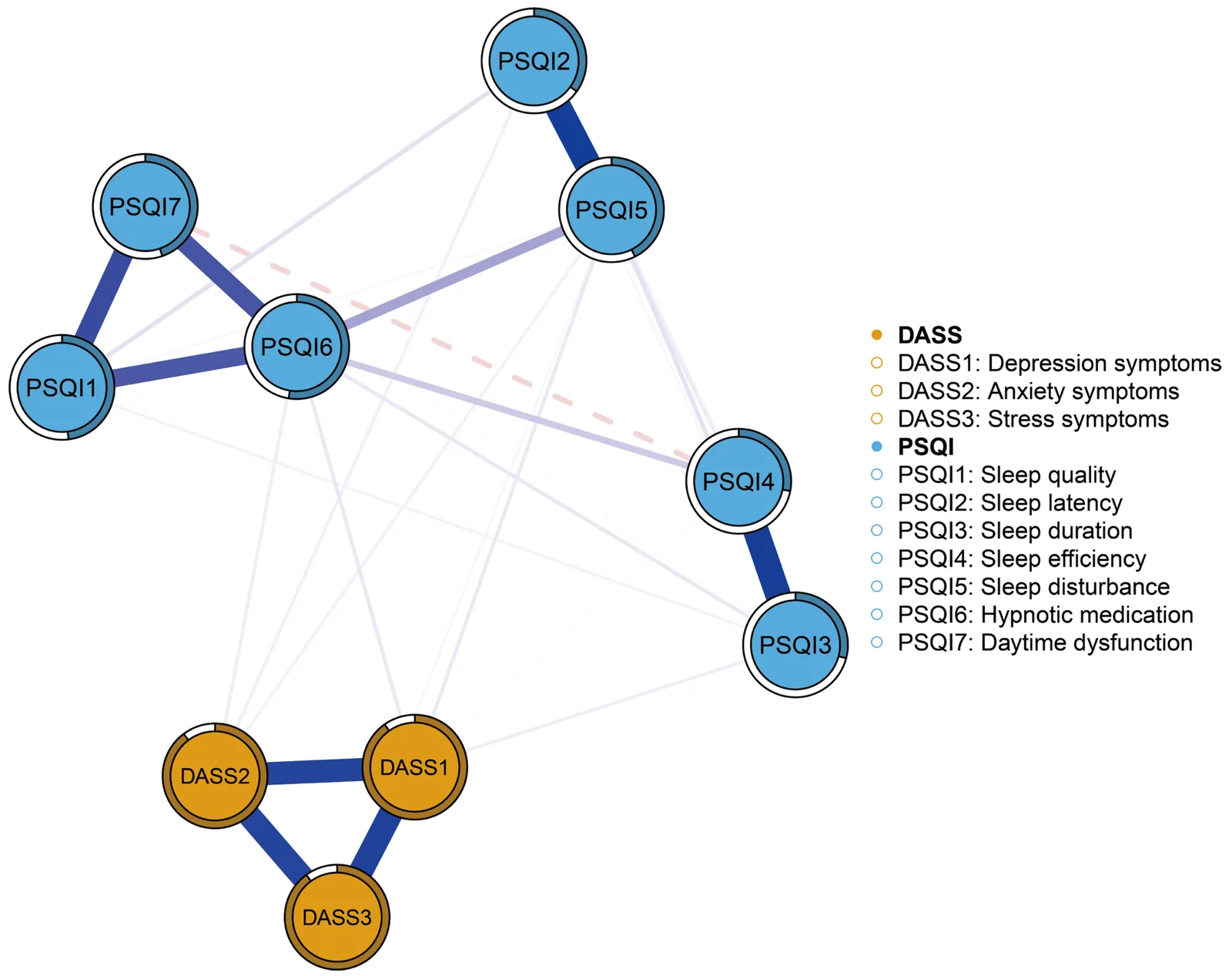
The component-level regularized partial-correlation network of DASS and PSQI dimensions. Note: The orange nodes denote the three DASS subscales and the blue nodes denote the seven PSQI components. The blue solid edges are positive and the red dashed edges are negative; the width and saturation indicate absolute edge magnitude. The ring around each node represents predictability (R2), or the proportion of variance explained by adjacent nodes.

**Figure 2:**
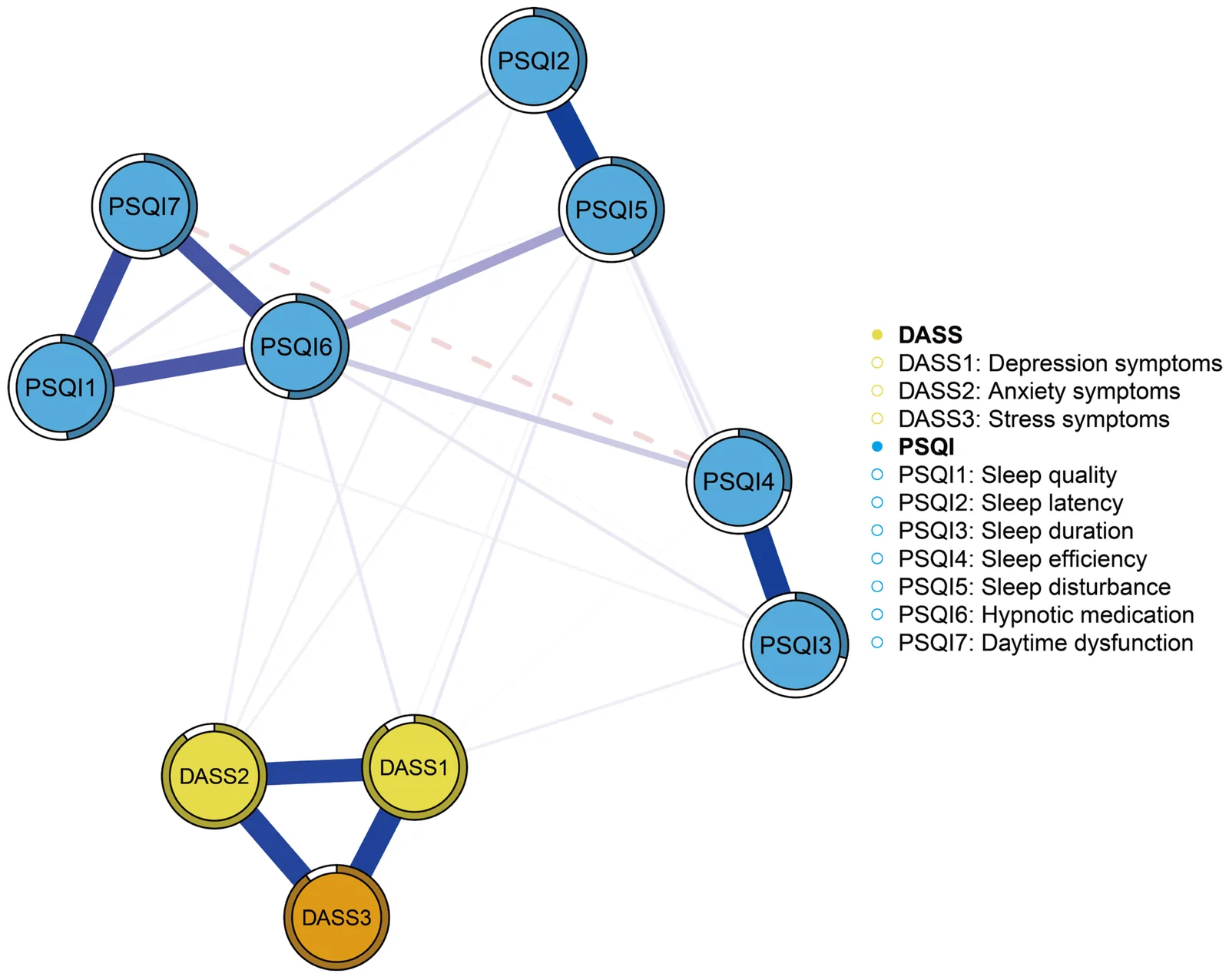
Standardized bridge-strength centrality in the DASS–PSQI network. Each labeled point represents a network node corresponding to a DASS subscale or PSQI component. The position of each point indicates its z-standardized bridge-strength value, and the lines connecting the plotted estimates are included only to facilitate visual comparison across nodes; they do not represent network edges or associations between nodes. Note: The values are z-standardized for visualization; the exact unstandardized bridge-strength estimates are reported in Table S2a. Higher values indicate greater absolute cross-domain connectivity and do not establish direction, causality, or clinical importance.

**Figure 3:**
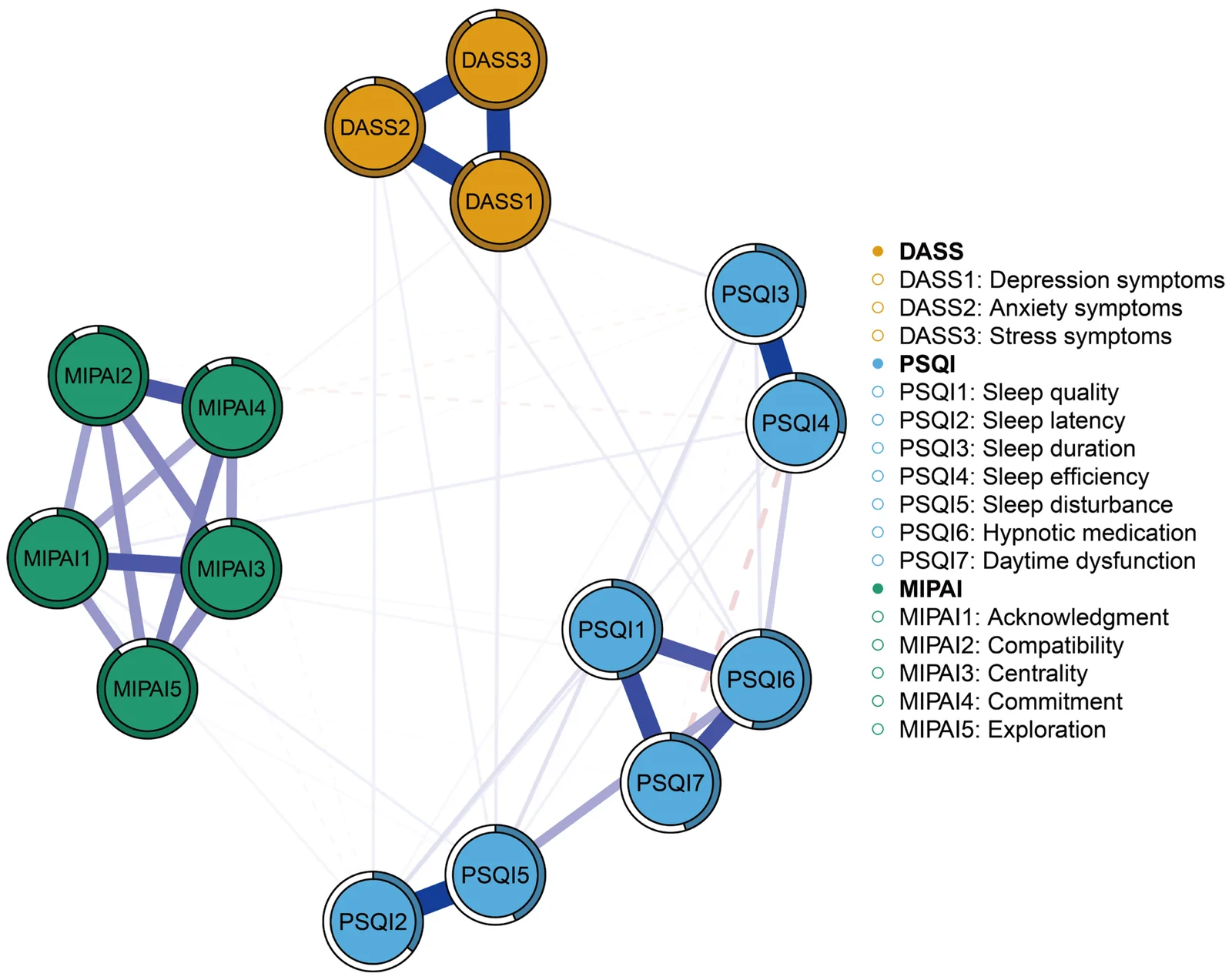
The component-level regularized partial-correlation network of DASS, PSQI, and MIPAI dimensions. Note: The orange, blue, and green nodes denote DASS subscales, PSQI components, and MIPAI dimensions, respectively. The blue solid edges are positive and the red dashed edges are negative; the width and saturation indicate absolute edge magnitude. The ring around each node represents predictability (R^2^).

**Figure 4:**
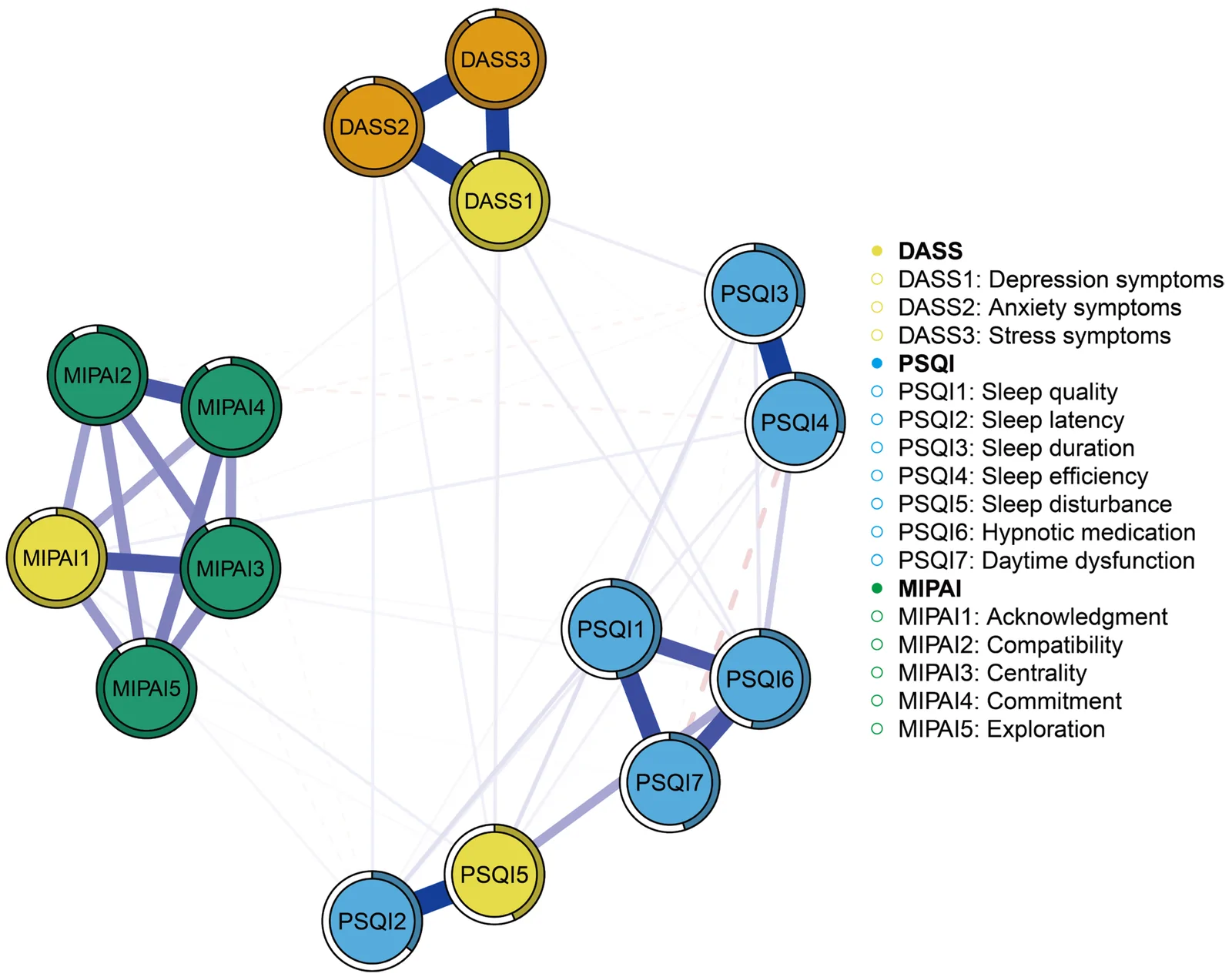
Standardized bridge-strength centrality in the DASS–PSQI network. Each labeled point represents a network node corresponding to a DASS subscale or PSQI component. The position of each point indicates its z-standardized bridge-strength value, and the lines connecting the plotted estimates are included only to facilitate visual comparison across nodes; they do not represent network edges or associations between nodes. Note: The values are z-standardized for visualization; the exact unstandardized bridge-strength estimates are reported in Table S2a. Higher values indicate greater absolute cross-domain connectivity and do not establish direction, causality, or clinical importance.

**Figure 5:**
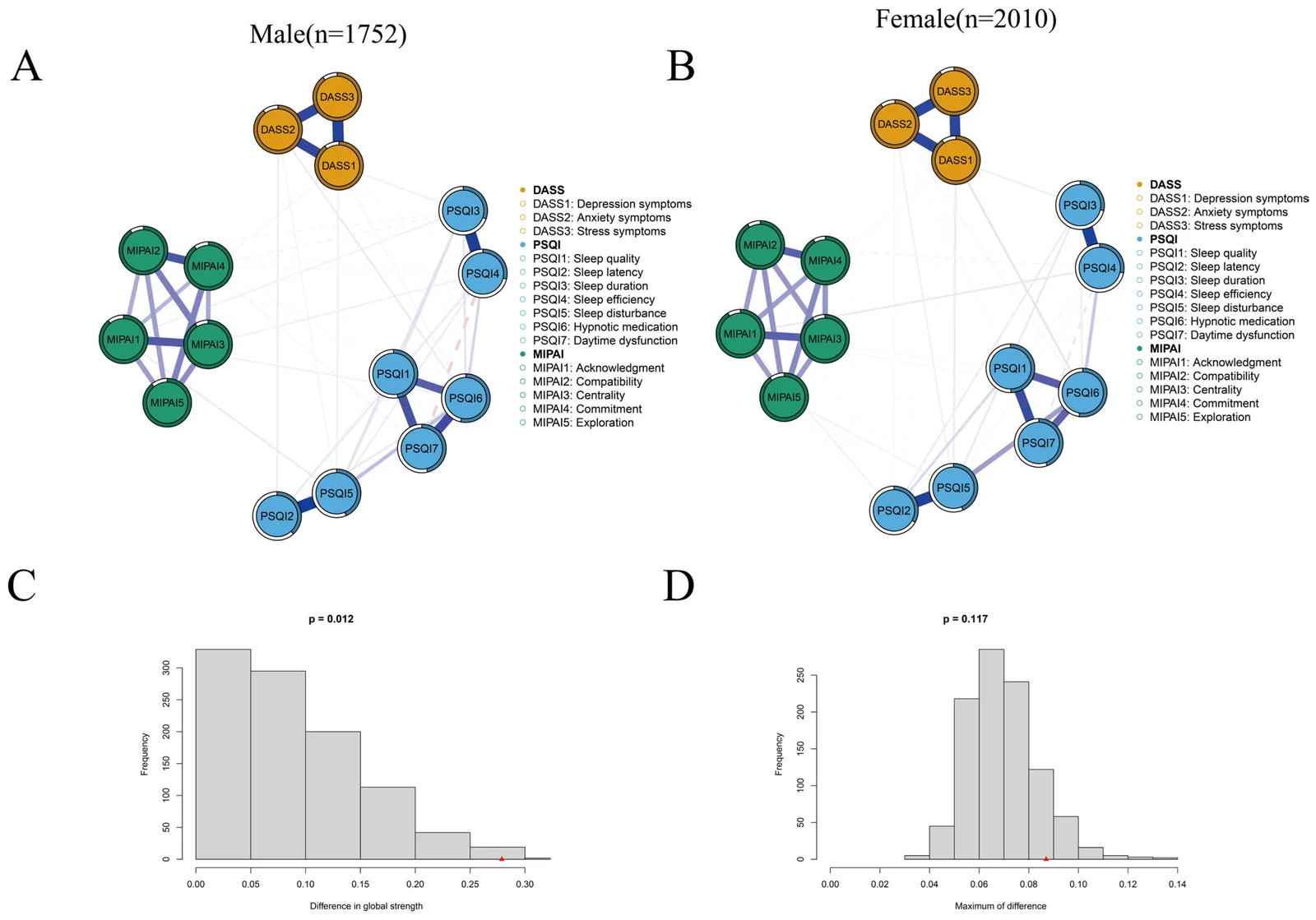
Sex-stratified three-domain networks and Network Comparison Test results for male and female participants. In panels (**A**,**B**), the nodes represent the DASS subscales, PSQI components, and MIPAI dimensions, with node colors indicating domain membership according to the legend. The lines represent regularized partial correlations between nodes after conditioning on all other variables in the network; line thickness reflects the absolute magnitude of the association, and line color indicates its direction. The absence of a line indicates that the corresponding edge was estimated as zero after regularization. Note: (**A**) Male network. (**B**) Female network. (**C**) Permutation distribution of the global-strength difference (male = 7.111; female = 6.832; *p* = 0.012). (**D**) Permutation distribution of the maximum network-structure difference (M = 0.087; *p* = 0.117). The red triangles indicate the observed statistics. The results are based on 1000 permutations.

**Table 1:** The baseline and demographic characteristics of the study sample (n = 3762).

Variables	Mean/N	SD/%
Age	20.26	3.45
Height (cm)	171.20	8.11
Weight (kg)	59.13	11.96
BMI (kg/m^2^)	20.31	4.58
**Sex**		
Male	1752	46.6
Female	2010	53.4
**Residence**		
Urban	1642	43.6
Rural	2120	56.4
**Family sibling status**		
Only child	2087	55.5
Non-only child	1675	44.5
**Smoking**		
Rarely or never	1480	39.3
Occasionally	828	22.0
Frequently or almost every day	1454	38.6
**E-cigarette use**		
Rarely or never	1538	40.9
Occasionally	708	18.8
Frequently or almost every day	1516	40.3
**Alcohol consumption**		
Rarely or never	1352	35.9
Occasionally	873	23.2
Frequently or almost every day	1537	40.9
**Coffee consumption**		
Rarely or never	1120	29.8
Occasionally	934	24.8
Frequently or almost every day	1708	45.4
**Tea consumption**		
Rarely or never	1074	28.5
Occasionally	760	20.2
Frequently or almost every day	1928	51.2
**Depression**		
Below cutoff (0–4)	903	24.0
Elevated (5–21)	2859	76.0
**Anxiety**		
Below cutoff (0–3)	771	20.5
Elevated (4–21)	2991	79.5
**Stress**		
Below cutoff (0–7)	1399	37.2
Elevated (8–21)	2363	62.8
**Sleep problem**		
Normal sleep (0–7)	743	19.8
Poor sleep quality (8–21)	3019	80.2

Note: The coding, category labels, frequencies, and percentages for smoking, e-cigarette use, and alcohol consumption were rechecked against the original questionnaire and confirmed to be correct. These variables represent self-reported frequency categories.

## Data Availability

De-identified analytic data and the analysis code are available on reasonable request from the authors.

## Supplementary Materials

The following supporting information can be downloaded at https://www.mdpi.com/article/10.3390/bs16101751/s1, Figure S1: Post hoc simulation-based sensitivity analysis for the integrated DASS–PSQI–MIPAI network; Figure S2: Standardized strength and bridge-strength centrality for the DASS-PSQI component-level network; Figure S3: Stability and accuracy of the DASS-PSQI network based on 1000 bootstrap resamples; Figure S4: Strength and bridge-strength analyses for the MIPAI–DASS network; Figure S5: Strength and bridge-strength analyses for the MIPAI–PSQI network; Figure S6: Standardized centrality for the DASS-PSQI-MIPAI component-level network; Figure S7: The stability and accuracy of the three-domain network based on 1000 bootstrap resamples; Figure S8: Residence-stratified networks and exploratory residence-based Network Comparison Test results; Table S1: Item-to-component mapping; Table S2a,b: Descriptive statistics, unstandardized centrality, one-step and two-step signed bridge expected influence, and predictability; Table S3: Estimated regularized partial-correlation adjacency matrix; Table S4: Network-specific MIPAI cross-domain metrics; Table S5: Non-zero between-community edges; Table S6a: Mixed graphical model sensitivity results; Table S6b: MIPAI bridge-strength rankings and MIPAI1 cross-domain edges; and Table S7: Sex-specific means and standard deviations for the 15 network variables.

## Abbreviations

BDNF	Brain-Derived Neurotrophic Factor
BMI	Body Mass Index
CI	Confidence Interval
CS-C	Correlation-Stability Coefficient
DASS-21	Depression Anxiety Stress Scales-21
EBICglasso	Extended Bayesian Information Criterion Graphical Least Absolute Shrinkage and Selection Operator
GGM	Graphical Gaussian Model
IP	Internet Protocol
LASSO	Least Absolute Shrinkage and Selection Operator
MIPAI-25	Multidimensional Inventory of Physical Activity Identity-25
NCT	Network Comparison Test
PSQI	Pittsburgh Sleep Quality Index
SD	Standard Deviation
WHO	World Health Organization
