# Supplementary material for: A Component-Level Network Analysis of Emotional Symptoms, Sleep Problems, and Physical Activity Identity at a Chinese University
Source: Behav Sci (Basel). 2026 Sep 25;16(10):1751. doi: 10.3390/bs16101751 (PMC13701230; doi:10.3390/bs16101751)
Supplement: Supplementary file 1 [file behavsci-16-01751-s001.zip › behavsci-4461199-supplementary.pdf]

Supplementary Material

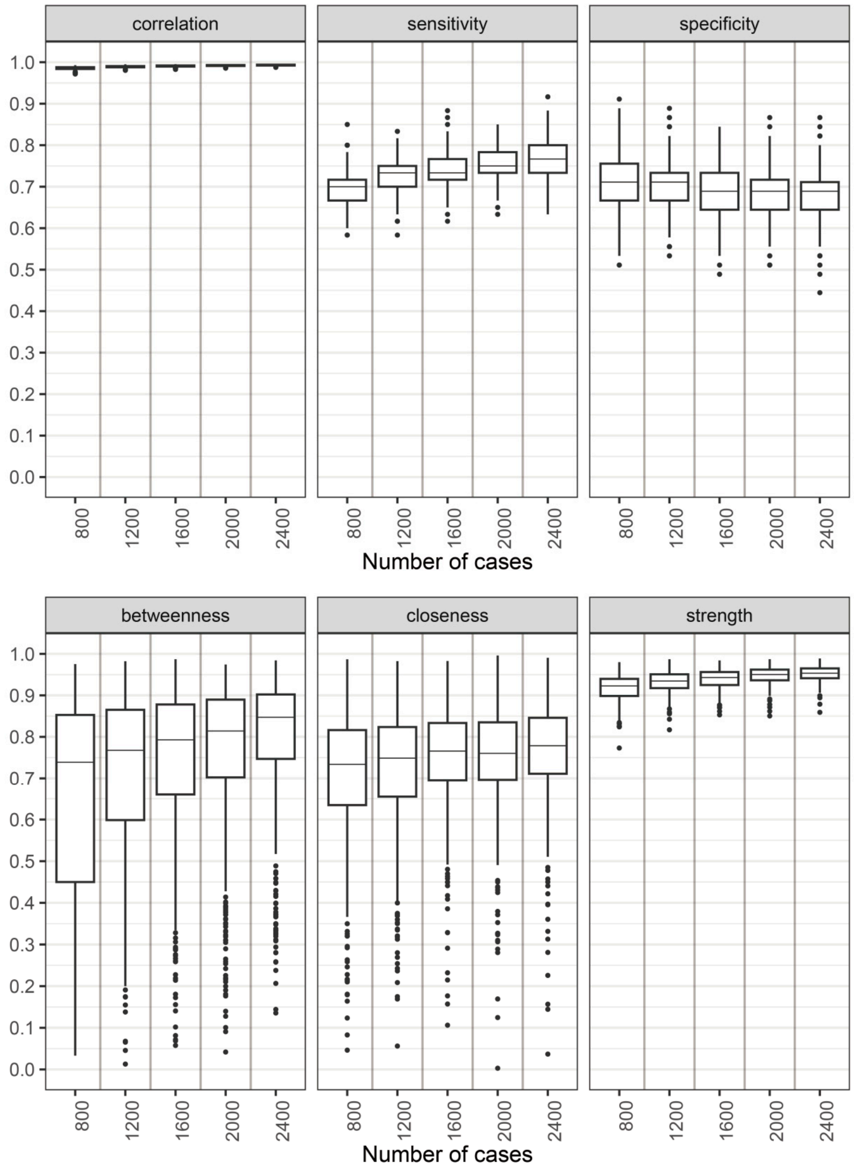

Figure S1. Post hoc simulation-based sensitivity analysis for the integrated DASS-PSQI-MIPAI network.

Note: The estimated 15-node network was used as the data-generating network, with five-level ordinal data simulated at sample sizes of 800, 1,200, 1,600, 2,000, and 2,400 and 500 replications per condition. Networks were re-estimated using netSimulator with EBICglasso and an EBIC tuning parameter of gamma = 0.50. The upper panels show the correlation between generating and re-estimated edge weights, sensitivity, and specificity. Sensitivity represents the proportion of non-zero edges correctly recovered, whereas specificity represents the proportion of zero edges correctly omitted. The lower panels show correlations between generating and re-estimated betweenness, closeness, and strength centrality estimates. Because the simulation was conducted after data collection and used a tuning setting different from the primary analysis, it is descriptive and is not interpreted as an a priori power or sample-size justification.

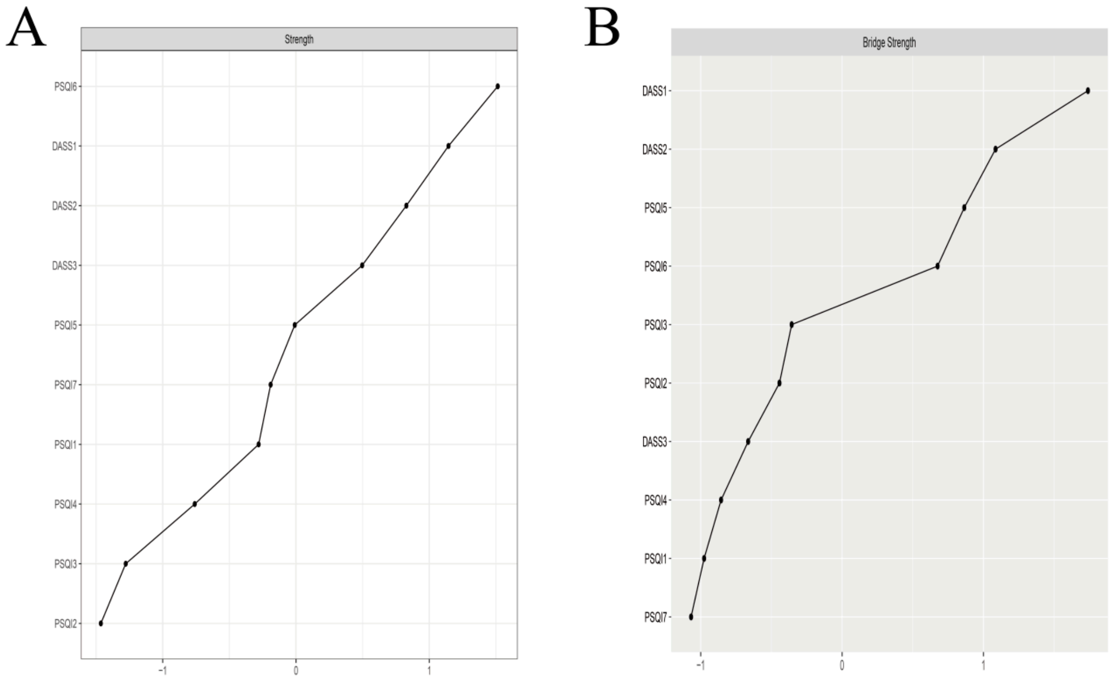

Figure S2. Standardized strength and bridge strength centrality indices for the DASS–PSQI network among study participants (z-scores). (A) Strength centrality. (B) Bridge strength centrality.

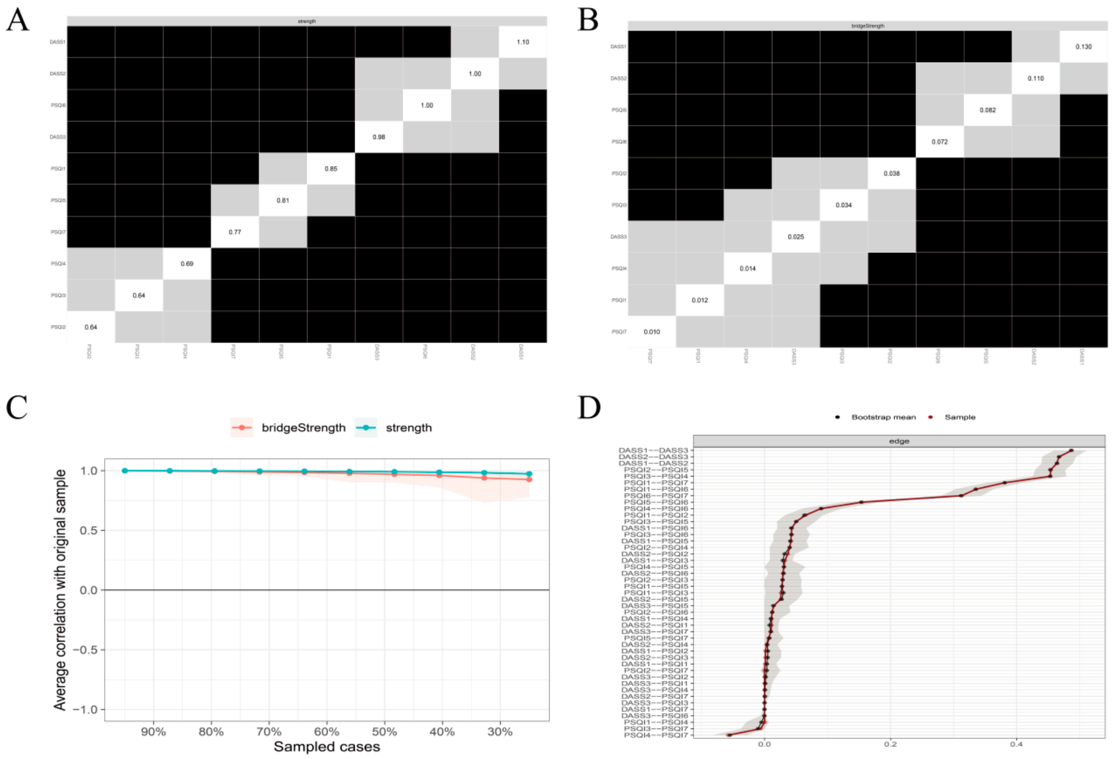

Figure S3. Stability and accuracy assessments for the DASS–PSQI network in the study sample. (A) Bootstrapped difference tests (1,000 resamples) for node strength centrality. (B) Nonparametric bootstrapped difference test for bridge strength. Gray cells indicate non-significant differences ($\alpha = 0.05$), whereas black cells indicate significant differences; diagonal white cells display the original point estimates. (C) Case-dropping bootstrap evaluation of the stability of node strength and bridge strength. The x-axis denotes the proportion of cases removed, and the y-axis denotes Pearson correlations between centrality estimates from the original network and those from re-estimated networks. Solid line = strength; dashed line = bridge strength; CS $\geq$ 0.50 indicates acceptable stability. (D) Bootstrapped 95% confidence intervals (CIs) for estimated edges. Red lines indicate sample estimates, and gray bands denote 95% CIs based on 1,000 bootstrap resamples. Edge labels for DASS–PSQI node pairs are listed along the right-hand y-axis.

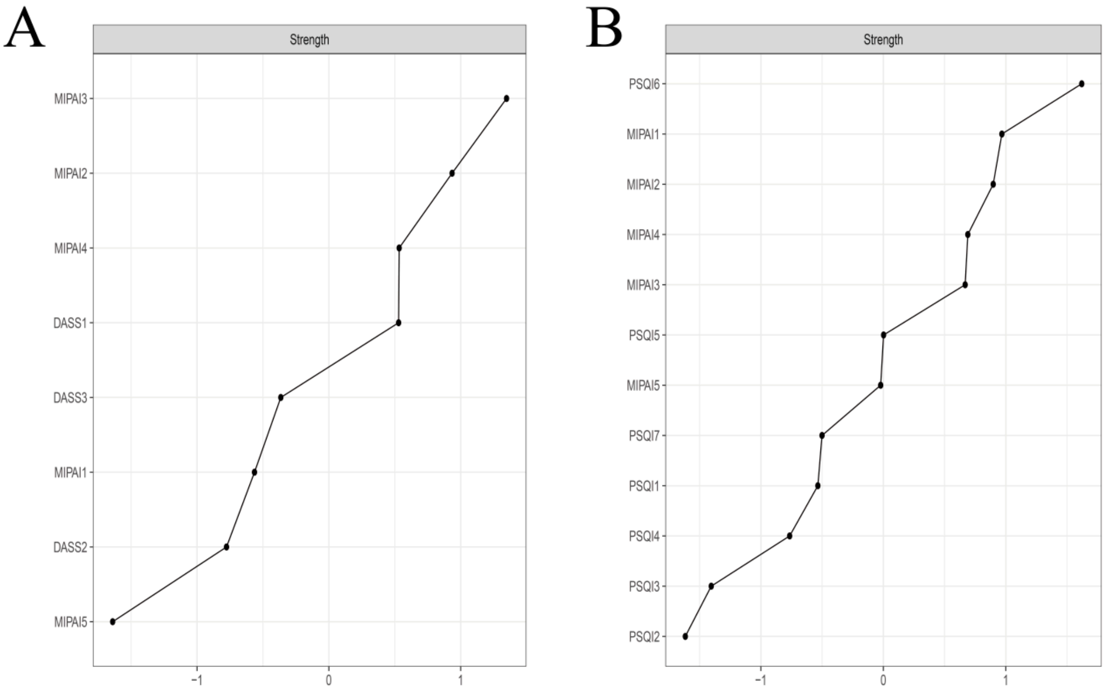

Figure S4. Standardized strength centrality of links between MIPAI and (A) DASS subscales and (B) PSQI components (z-scores).

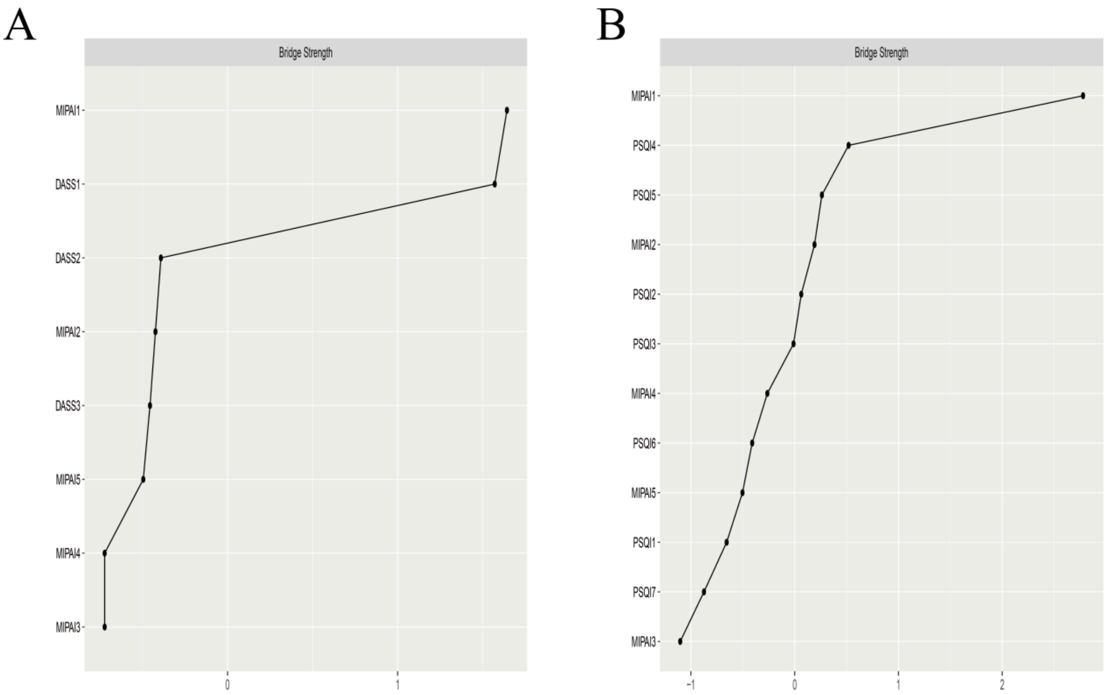

Figure S5. Standardized bridge strength centrality of links between MIPAI and (A) DASS subscales and (B) PSQI components (z-scores).

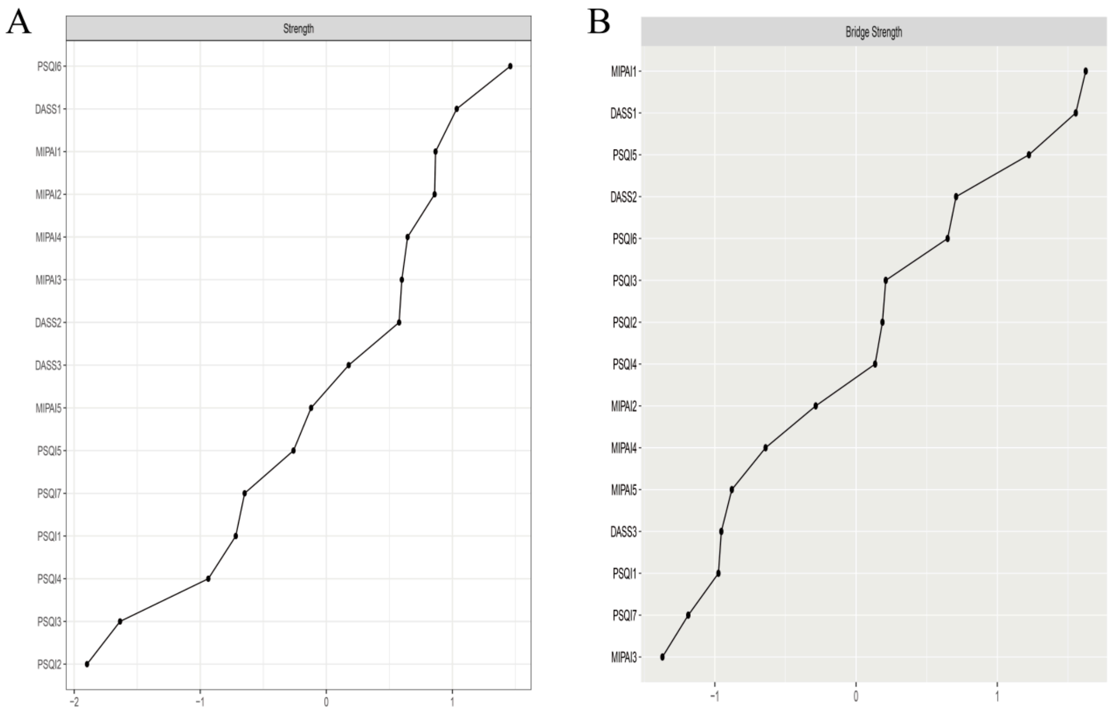

Figure S6. Standardized strength and bridge strength centrality indices for the integrated DASS, PSQI, and MIPAI network among study participants (z-scores). (A) Strength centrality. (B) Bridge strength centrality.

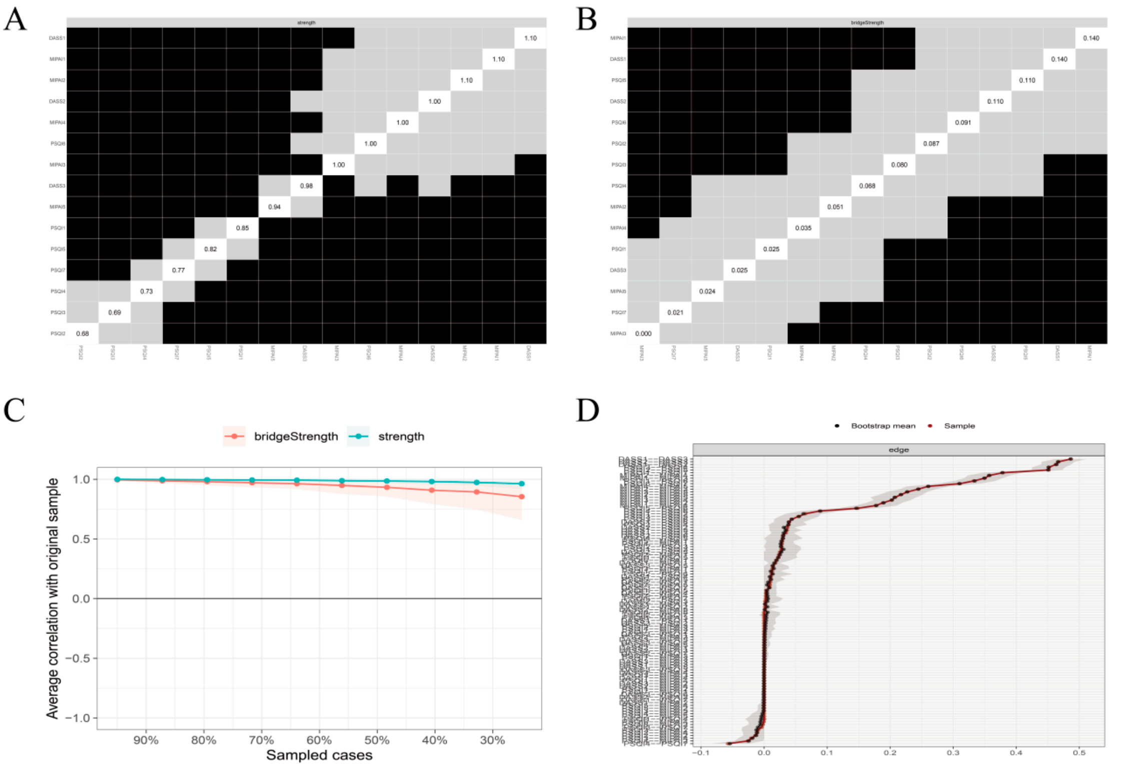

Figure S7. Stability and accuracy assessments for the integrated DASS, PSQI, and MIPAI network in the study sample. (A) Bootstrapped difference tests (1,000 resamples) for node strength centrality. (B) Nonparametric bootstrapped difference test for bridge strength. Gray cells indicate non-significant differences ($\alpha = 0.05$), whereas black cells indicate significant differences; diagonal white cells display the original point estimates. (C) Case-dropping bootstrap evaluation of the stability of node strength and bridge strength. The x-axis denotes the proportion of cases removed, and the y-axis denotes Pearson correlations between centrality estimates from the original network and those from re-estimated networks. Solid line = strength; dashed line = bridge strength; CS $\geq$ 0.50 indicates acceptable stability. (D) Bootstrapped 95% confidence intervals (CIs) for estimated edges. Red lines indicate sample estimates, and gray bands denote 95% CIs based on 1,000 bootstrap resamples. Edge labels for DASS–PSQI–MIPAI node pairs are listed along the right-hand y-axis. The edge-weight CS coefficient reported in the manuscript was calculated from the same case-dropping bootstrap object but is not shown in Panel C; it summarizes correlations of the complete edge-weight vector rather than individual-edge confidence intervals.

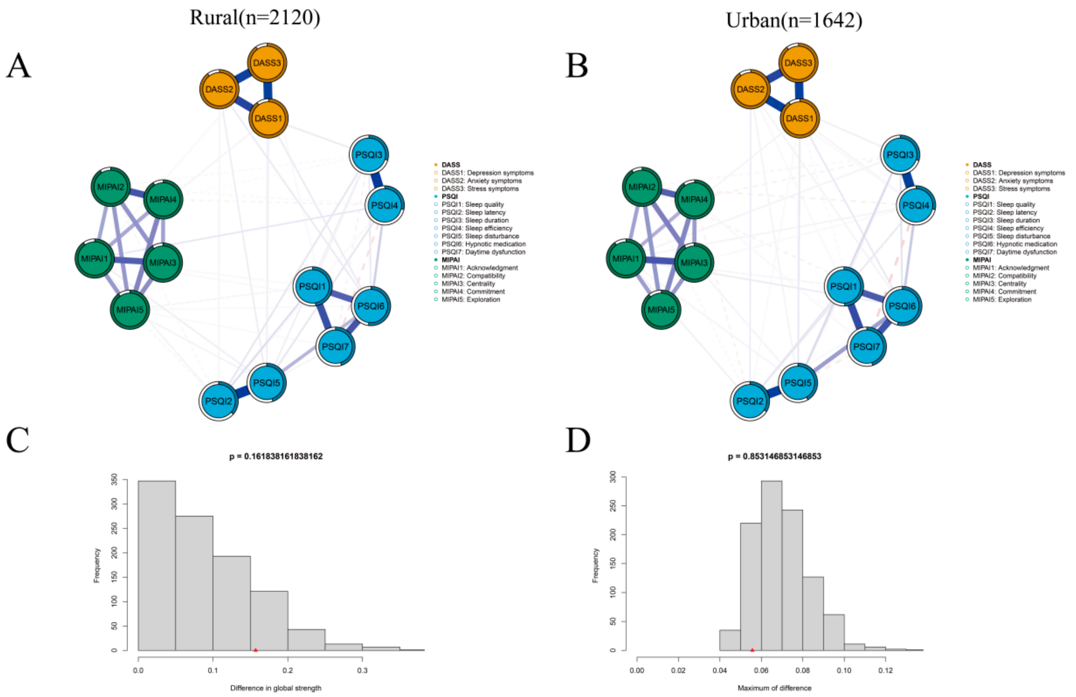

Figure S8. Residence-stratified three-domain networks and Network Comparison Test results for rural and urban participants. Note: (A) Rural network (n = 2,120). (B) Urban network (n = 1,642). Orange, blue, and green nodes denote DASS subscales, PSQI components, and MIPAI dimensions, respectively. Blue solid edges indicate positive associations, red dashed edges indicate negative associations, and edge width/saturation reflects absolute edge magnitude. The ring around each node represents predictability. (C) Permutation distribution for the global-strength difference between rural and urban networks (P = 0.162). (D) Permutation distribution for the maximum network-structure difference (P = 0.853). Red triangles indicate the observed statistics. Results are based on 1,000 permutations.

Table S1. Item-level mapping of the three primary modules—depression (DASS1), anxiety (DASS2), and stress (DASS3)—as well as sleep problems (PSQI) and physical activity identity (MIPAI) among study participants.

Scale	Abbr	Symptoms/Dimensions	Items
DASS	DASS1	Depression symptoms	3. I could not seem to experience any positive feeling at all.
			5. I found it difficult to work up the initiative to do things.
			10. I felt that I had nothing to look forward to.
			13. I felt down-hearted and blue.
			16. I was unable to become enthusiastic about anything.
			17. I felt I was not worth much as a person.
			21. I felt that life was meaningless.
	DASS2	Anxiety symptoms	2. I was aware of dryness of my mouth.
			4. I experienced breathing difficulty (e.g., excessively rapid breathing, breathlessness in the absence of physical exertion).
			7. I experienced trembling (e.g., in the hands).
			9. I was worried about situations in which I might panic and make a fool of myself.
			15. I felt I was close to panic.
			19. I was aware of the action of my heart in the absence of physical exertion (e.g., sense of heart rate increase, heart missing a beat).
			20. I felt scared without any good reason.
	DASS3	Stress symptoms	1. I found it hard to wind down.
			6. I tended to over-react to situations.
			8. I felt that I was using a lot of nervous energy.
			11. I found myself getting agitated.
			12. I found it difficult to relax.

			14. I was intolerant of anything that kept me from getting on with what I was doing.
			18. I found that I was rather touchy.
PSQI	PSQI1	Sleep Quality	6. During the past month, how would you rate your sleep quality overall?
	PSQI2	Sleep Latency	2. During the past month, how long (in minutes) has it usually taken you to fall asleep each night?
			5a. During the past month, cannot get to sleep within 30 minutes.
	PSQI3	Sleep Duration	4. During the past month, how many hours of actual sleep did you get at night?
	PSQI4	Sleep Efficiency	1. During the past month, when have you usually gone to bed at night?
			2. During the past month, how long (in minutes) has it usually taken you to fall asleep each night?
			4. During the past month, how many hours of actual sleep did you get at night?
	PSQI5	Sleep Disturbance	5b. Wake up in the middle of the night or early morning.
			5c. Have to get up to use the bathroom.
			5d. Cannot breathe comfortably.
			5e. Cough or snore loudly.
			5f. Feel too cold.
			5g. Feel too hot.
			5h. Have bad dreams.
			5i. Have pain.
			5j. Other reasons disturbing sleep.
	PSQI6	Hypnotic Medication	7. During the past month, how often have you taken medicine to help you sleep?
	PSQI7	Daytime Dysfunction	8. During the past month, how often have you had trouble staying awake while driving, eating meals, or engaging in social activity?
			9. During the past month, how much of a problem has it been for you to keep up enough enthusiasm to get things done?
MIPAI	MIPAI 1	Acknowledgment	1. My relatives would say I am someone who is physically active on a regular basis.
			5. Others see me as someone who engages in physical activity regularly.

			10. Compared to others my age, I am a physically active individual.
			16. My friends would say I am someone who is physically active on a regular basis.
			25. Other people who are physically active would say I am someone who is physically active on a regular basis.
	MIPAI 2	Compatibility	6. Being physically active is compatible with aspects of my life that are important to me (e.g., family, spirituality, school, work, social relations, etc.).
			8. Being physically active is something that fits the way I want to live.
			9. Being physically active is compatible with how I see myself.
			14. Being physically active is something that fits who I am.
			23. Being physically active is compatible with what I am doing in other areas of my life.
	MIPAI 3	Centrality	2. I see myself as an exerciser.
			13. Others view being physically active as part of my personality.
			19. I am someone who keeps in shape.
			22. Being physically active is part of "who I am."
			24. I see myself as a physically active person.
	MIPAI 4	Commitment	3. I need to be physically active to feel good about myself.
			7. I am committed towards being physically active.
			11. I really enjoy being physically active.
			12. Physical activity is something I think about often.
			15. I feel good about engaging in a physically active lifestyle.
	MIPAI 5	Exploration	4. I talk with other people about how physically active I am.
			17. In order to learn more about physical activity, I have often talked to other people who I consider to be physically active.
			18. I have spent time trying to find out more about physical activity, such as what, how and where to do different activities.

			20. I try to find out a lot about physical activity.
			21. I make a lot of effort to keep finding out new things about physical activity.

Instrument-language note: Published studies support the psychometric properties of Chinese-language DASS-21 and PSQI instruments; however, the retained study materials did not document the translator or exact source edition administered. The MIPAI-25 Chinese wording was prepared internally by the research team specifically for this survey. No formal back-translation, independent expert review, cognitive interviewing, or pilot testing was conducted, and separately documented written permission to translate and use the instrument could not be confirmed from the retained study records. The Chinese MIPAI-25 wording has not been independently validated. The item wording below is provided for transparency and should not be interpreted as a validated Chinese adaptation.

Table S2a. Descriptive statistics, centrality metrics, one-step and two-step signed bridge expected influence, and node predictability for depression (DASS1), anxiety (DASS2), stress (DASS3), and sleep problems (PSQI).

Node	Mean	SD	Skewness	Kurtosis	Strength	Bridge strength	Signed bridge expected influence (1-step)	Signed bridge expected influence (2-step)	Predictability
DASS1	10.191	6.431	-0.073	-1.242	1.070	0.121	0.121	0.274	0.902
DASS2	10.289	6.374	-0.066	-1.237	1.022	0.093	0.093	0.235	0.898
DASS3	10.358	6.302	-0.094	-1.198	0.972	0.018	0.018	0.134	0.900
PSQI1	1.561	1.008	-0.032	-1.089	0.854	0.004	0.004	0.038	0.480
PSQI2	1.666	0.819	-0.228	-0.434	0.675	0.027	0.027	0.099	0.349
PSQI3	1.632	0.944	-0.13	-0.894	0.703	0.031	0.031	0.074	0.291
PSQI4	1.697	1.178	-0.236	-1.452	0.781	0.010	0.010	0.046	0.280
PSQI5	1.847	0.917	-0.157	-1.038	0.895	0.083	0.083	0.192	0.426
PSQI6	1.395	1.12	0.066	-1.376	1.126	0.075	0.075	0.165	0.525
PSQI7	1.818	0.955	-0.321	-0.882	0.868	0.001	0.001	0.029	0.447

Table S2b. Descriptive statistics, centrality metrics, one-step and two-step signed bridge expected influence, and node predictability for depression (DASS1), anxiety (DASS2), stress (DASS3), sleep problems (PSQI), and MIPAI in the study sample.

Node	Mean	SD	Skewness	Kurtosis	Strength	Bridge strength	Signed bridge expected influence (1-step)	Signed bridge expected influence (2-step)	Predictability
DASS1	10.191	6.431	-0.073	-1.242	1.078	0.130	0.130	0.295	0.902
DASS2	10.289	6.374	-0.066	-1.237	1.021	0.093	0.093	0.241	0.898
DASS3	10.358	6.302	-0.094	-1.198	0.972	0.019	0.019	0.141	0.900
PSQI1	1.561	1.008	-0.032	-1.089	0.860	0.018	0.018	0.071	0.481
PSQI2	1.666	0.819	-0.228	-0.434	0.714	0.069	0.023	0.107	0.353
PSQI3	1.632	0.944	-0.13	-0.894	0.746	0.070	0.015	0.046	0.294
PSQI4	1.697	1.178	-0.236	-1.452	0.833	0.067	0.013	0.044	0.283
PSQI5	1.847	0.917	-0.157	-1.038	0.917	0.116	0.116	0.254	0.433
PSQI6	1.395	1.12	0.066	-1.376	1.131	0.090	0.088	0.203	0.528
PSQI7	1.818	0.955	-0.321	-0.882	0.869	0.008	0.008	0.054	0.450
MIPAI1	22.032	7.711	-0.285	-0.752	1.057	0.134	0.134	0.234	0.902
MIPAI2	22.436	7.554	-0.342	-0.648	1.056	0.048	-0.048	-0.060	0.912
MIPAI3	22.207	7.557	-0.299	-0.681	1.024	0.000	0.000	0.034	0.913
MIPAI4	22.347	7.598	-0.354	-0.648	1.030	0.033	-0.028	-0.036	0.910
MIPAI5	22.238	7.558	-0.31	-0.68	0.935	0.022	0.022	0.050	0.896

Note: Strength and bridge strength are unstandardized sums of absolute regularized edge weights. Signed bridge expected influence retains edge signs; one-step values summarize direct between-community edges, whereas two-step values additionally incorporate indirect paths through adjacent nodes. Predictability is the proportion of variance explained by adjacent nodes and is interpreted descriptively.

Table S3. Estimated regularized partial-correlation adjacency matrix for DASS subscales, PSQI components, and MIPAI dimensions.

	DASS1	DASS2	DASS3	PSQI1	PSQI2	PSQI3	PSQI4	PSQI5	PSQI6	PSQI7	MIPAI1	MIPAI2	MIPAI3	MIPAI4	MIPAI5
DASS1	0.000														
DASS2	0.462	0.000													
DASS3	0.486	0.467	0.000												
PSQI1	0.000	0.002	0.000	0.000											
PSQI2	0.000	0.028	0.000	0.059	0.000										
PSQI3	0.034	0.000	0.000	0.024	0.015	0.000									
PSQI4	0.007	0.002	0.000	0.000	0.034	0.522	0.000								
PSQI5	0.035	0.025	0.017	0.006	0.537	0.058	0.025	0.000							
PSQI6	0.038	0.034	0.000	0.350	0.000	0.047	0.103	0.176	0.000						
PSQI7	0.000	0.000	0.000	0.404	0.000	-0.010	-0.082	0.000	0.365	0.000					
MIPAI1	0.017	0.000	0.002	0.011	0.018	0.009	0.031	0.028	0.017	0.000	0.000				
MIPAI2	0.000	0.000	0.000	0.000	-0.013	-0.008	-0.027	0.000	-0.001	0.000	0.189	0.000			
MIPAI3	0.000	0.000	0.000	0.000	0.000	0.000	0.000	0.000	0.000	0.000	0.349	0.244	0.000		
MIPAI4	0.000	0.000	0.000	0.000	-0.010	-0.020	0.000	0.000	-0.000	0.002	0.178	0.357	0.203	0.000	
MIPAI5	0.000	0.001	0.000	0.004	0.000	0.000	0.000	0.011	0.000	0.006	0.208	0.217	0.228	0.260	0.000

Note: Diagonal cells are fixed to zero; off-diagonal zero values indicate edges shrunk to zero by EBICglasso regularization rather than ordinary bivariate correlations.

Table S4. Network-specific MIPAI strength and bridge-strength metrics in the MIPAI-DASS and MIPAI-PSQI two-domain networks. Note: Each column was calculated within the named two-domain network and is not directly interchangeable with the overall three-domain bridge-strength value in Table S2b. MIPAI = Multidimensional Inventory of Physical Activity Identity; DASS = Depression Anxiety Stress Scales; PSQI = Pittsburgh Sleep Quality Index.

MIPAI dimension	Strength (MIPAI-DASS)	Strength (MIPAI-PSQI)	Bridge strength (MIPAI-DASS)	Bridge strength (MIPAI-PSQI)
MIPAI1	0.957	1.063	0.033	0.138
MIPAI2	1.012	1.054	0.004	0.046
MIPAI3	1.027	1.024	0.000	0.000
MIPAI4	0.997	1.027	0.000	0.030
MIPAI5	0.918	0.935	0.003	0.021

Table S5. Non-zero between-community regularized edge weights in the primary DASS-PSQI-MIPAI network, ordered by absolute magnitude.

Node 1	Node 2	Edge weight	Direction	Absolute weight
DASS1	PSQI6	0.0383508486966764	Positive	0.0383508486966764
DASS1	PSQI5	0.0346165458513601	Positive	0.0346165458513601
DASS2	PSQI6	0.0340786383891966	Positive	0.0340786383891966
DASS1	PSQI3	0.0336663981816462	Positive	0.0336663981816462
PSQI4	MIPAI1	0.0314218528956924	Positive	0.0314218528956924
PSQI5	MIPAI1	0.0281677303452653	Positive	0.0281677303452653
DASS2	PSQI2	0.0280156705884065	Positive	0.0280156705884065
PSQI4	MIPAI2	-0.0268768195380616	Negative	0.0268768195380616
DASS2	PSQI5	0.0254035670424482	Positive	0.0254035670424482
PSQI3	MIPAI4	-0.0199094158414391	Negative	0.0199094158414391
PSQI2	MIPAI1	0.0181622330808367	Positive	0.0181622330808367
DASS1	MIPAI1	0.0171876231132674	Positive	0.0171876231132674
DASS3	PSQI5	0.0169246242297295	Positive	0.0169246242297295
PSQI6	MIPAI1	0.0166321471571824	Positive	0.0166321471571824
PSQI2	MIPAI2	-0.012976485260086	Negative	0.012976485260086
PSQI1	MIPAI1	0.0113442084156034	Positive	0.0113442084156034
PSQI5	MIPAI5	0.0105378617696234	Positive	0.0105378617696234
PSQI2	MIPAI4	-0.0102693125015447	Negative	0.0102693125015447
PSQI3	MIPAI1	0.00899199585960321	Positive	0.00899199585960321
PSQI3	MIPAI2	-0.00790010039042424	Negative	0.00790010039042424
DASS1	PSQI4	0.00660791581568089	Positive	0.00660791581568089

Node 1	Node 2	Edge weight	Direction	Absolute weight
PSQI7	MIPAI5	0.0060943232805009	Positive	0.0060943232805009
PSQI1	MIPAI5	0.00431680744973332	Positive	0.00431680744973332
DASS2	PSQI4	0.00220493079999531	Positive	0.00220493079999531
PSQI7	MIPAI4	0.00206242252450053	Positive	0.00206242252450053
DASS2	PSQI1	0.002014676237955	Positive	0.002014676237955
DASS3	MIPAI1	0.0016617832254843	Positive	0.0016617832254843
DASS2	MIPAI5	0.000951957360822929	Positive	0.000951957360822929
PSQI6	MIPAI2	-0.000618381510371452	Negative	0.000618381510371452
PSQI6	MIPAI4	-0.000308990123980076	Negative	0.000308990123980076

Note: Positive and negative signs describe conditional association direction and do not establish benefit, harm, or causality.

Table S6a. Agreement between the primary EBICglasso network and the mixed graphical model sensitivity network.

Metric	Value
Absolute edge-weight Spearman correlation	0.727
Strength-rank Spearman correlation	-0.293
Bridge-strength-rank Spearman correlation	0.489
Primary EBICglasso nonzero edges	60
MGM nonzero edges	86

Note: DASS and MIPAI summed scores were modeled as Gaussian and PSQI components as four-level categorical variables; EBIC regularization used lambdaGam = 0.25 and the OR rule. Agreement metrics compare the sensitivity model with the primary EBICglasso network. Edge-weight agreement is based on absolute edge weights because signs are not consistently defined for all mixed-variable MGM interactions.

Table S6b. MIPAI bridge-strength rankings in the mixed graphical model sensitivity analysis.

Node	MGM bridge strength	Rank among MIPAI dimensions
MIPAI1	0.390	1
MIPAI4	0.307	2
MIPAI2	0.274	3
MIPAI5	0.226	4
MIPAI3	0.198	5

Table S6c. Non-zero MIPAI1 cross-domain edges retained in the mixed graphical model sensitivity analysis.

Edge	Domain	Absolute MGM edge weight
MIPAI1-PSQI2	PSQI	0.082
MIPAI1-PSQI1	PSQI	0.062
MIPAI1-PSQI7	PSQI	0.057
MIPAI1-PSQI3	PSQI	0.052
MIPAI1-PSQI6	PSQI	0.050
MIPAI1-PSQI4	PSQI	0.040
MIPAI1-PSQI5	PSQI	0.038
MIPAI1-DASS1	DASS	0.008

Note: Edge weights in Table S6c are absolute MGM edge weights. MIPAI1 retained the largest bridge-strength point estimate among the MIPAI dimensions in the MGM sensitivity model.

Table S7. Sex-specific means and standard deviations for the 15 network variables.

**Node**	**Male, n = 1,752 Mean**	**Male SD**	**Female, n = 2,010 Mean**	**Female SD**
DASS1	10.624	6.391	9.813	6.442
DASS2	10.651	6.323	9.974	6.402
DASS3	10.739	6.289	10.026	6.296
PSQI1	1.558	1.022	1.563	0.995
PSQI2	1.684	0.832	1.650	0.807
PSQI3	1.648	0.961	1.619	0.929
PSQI4	1.723	1.200	1.675	1.159
PSQI5	1.864	0.920	1.832	0.914
PSQI6	1.449	1.112	1.348	1.126
PSQI7	1.840	0.938	1.800	0.969
MIPAI1	22.466	7.741	21.653	7.667
MIPAI2	22.922	7.561	22.011	7.524
MIPAI3	22.593	7.631	21.871	7.477
MIPAI4	22.845	7.576	21.913	7.592
MIPAI5	22.656	7.541	21.874	7.555

Note: Values are unadjusted descriptive statistics. Sex was coded as male or female in the retained dataset. Differences in score distributions may contribute to between-group network differences; the table is descriptive and does not establish measurement invariance.
